# Cutaneous gain-of-function mutation of LRIG3 leads to alopecia by upregulation of ERBB, PI3K/AKT, NOTCH1 signaling pathways

**DOI:** 10.1038/s41598-026-54295-5

**Published:** 2026-05-30

**Authors:** Saeedeh Ghorbanalipoor, Christine Hösl, Thomas Kolbe, Maik Dahlhoff

**Affiliations:** 1 Laboratory Animal Medicine, Department of Biological Sciences and Pathobiology, University of Veterinary Medicine Vienna, Veterinärplatz 1, 1210 Vienna, Austria; 2 Institute of Molecular Animal Breeding and Biotechnology, Gene Center, LMU München, Feodor-Lynen-Str. 25, 81377 Munich, Germany; 3 Department of Agricultural Sciences, University of Natural Resources and Life Sciences, 1180 Vienna, Austria

**Keywords:** LRIG3, Hair follicle, Skin, ERBB, NOTCH1, PI3K/AKT, Cell biology, Molecular biology

## Abstract

Homeostasis of the skin relies on meticulous regulation of integrated pathways. Within this network, ERBB receptors are of great importance in controlling the proliferation, differentiation, and survival of epidermal and hair follicle (HF) keratinocytes as well as sebaceous glands (SGs) in the skin. Consequently, leucine-rich repeats and immunoglobulin-like domains (LRIG) protein family comprising LRIG1, LRIG2, and LRIG3 as one of the fundamental regulators of ERBB receptors plays a pivotal role in tissue development and homeostasis. The function of LRIGs in maintaining skin homeostasis is primarily understood due to the most extensively studied member, LRIG1. However, the role of LRIG3, the last member of this protein family in the skin, is unknown. While its function has been demonstrated in development and maintenance of the homeostasis of various tissues such as inner ear and neural crest morphogenesis via modulating NOTCH and fibroblast growth factor signaling pathways, respectively. Here, we provided the first insights into how *Lrig3* overexpression in mouse skin, using the Tet-Off-System, influences cutaneous homeostasis. After overcoming the prenatal lethality of *Lrig3* overexpression and obtaining an adequate number of skin-specific *Lrig3* transgenic (*Lrig3*-TG) mice, the first clinical feature was hair loss in these mice. Subsequent investigations revealed extensive changes in the skin protein profile, particularly in keratinocyte differentiation as well as HF and SG markers, along with altered signaling pathways governing HF development. Our study implies up-regulation of the components of ERBB, NOTCH, PI3K/AKT pathways in the skin of *Lrig3*-TG mice, suggesting a potential role of the *Lrig3* in maintaining skin balance.

**Supplementary Information:**

The online version contains supplementary material available at https://doi.org/10.1038/s41598-026-54295-5.

## Introduction

The leucine-rich repeat and immunoglobulin-like domains (LRIG) proteins are a family of transmembrane proteins encompassing LRIG1, LRIG2, and LRIG3 whose main action is to attune the receptor tyrosine kinases (RTKs) including the ERBB family^1^. The normal activity of the ERBB network is a cornerstone to the well-balanced functioning of the epidermis, and its appendages. The epidermal growth factor EGFR/ERBB1/HER1^2–6^ and associated receptors, ERBB2/HER2/Neu^7–9^, ERBB3/HER3^7,8,10^, and ERBB4/HER4^11^ have been found to regulate key functions involved in epidermis, sebaceous gland (SG) and hair follicle (HF) development and homeostasis. Therefore, the LRIG family, as an important regulatory axis of ERBB signaling, is essential in preserving the skin balance. The expression of all LRIG proteins has been demonstrated in different compartments of the human skin^12^. LRIG1, the most well-studied LRIG family member in the skin, is known as a negative regulator of ERBB signaling, including EGFR^13^, ERBB2, ERBB3, and ERBB4^14^. This makes it a prerequisite for regulation of epidermal, HF, and SG stem cell quiescence and proliferation^15–17^. *Lrig1*-knockout mice render psoriasiform epidermal hyperplasia^18^, while skin-specific overexpression of LRIG1 leads to epidermal hyperplasia along with alopecia^19^. In contrast, cutaneous overexpression of LRIG2 has been shown to exert a positive regulatory role in the modulation of EGFR/ERBB signaling, resulting in accelerated skin tumorigenesis^20^. The function of LRIG3, the last member of this family, remains unclear in the context of skin homeostasis. However, it has been shown that LRIG3 is of critical importance in both the development and maintenance of homeostasis across various tissues. For instance, LRIG3 takes an essential role in proper inner ear formation, particularly by restricting the expression of netrin1, a component in NOTCH signaling, for correct semicircular canal development^21^. LRIG3 is also implicated in neural crest morphogenesis by modulating fibroblast growth factor (FGF) and wingless-type MMTV integration site family (Wnt) signaling pathways^22^. Furthermore, LRIG3 collaborates with LRIG1 to attune rearranged during transfection (RET) receptor signaling in sensory neuron development^23^. Additionally, LRIG3 is capable of inhibiting other RTKs like the FGF receptor and may partake in cardiac function, as its deficiency is linked with cardiac hypertrophy^24^. To obtain clues to the LRIG3 function in skin homeostasis and development, we established a mouse line that skin-specifically overexpresses LRIG3 using the Tet-Off system driven by the keratin 5 (*Krt5*) promoter. The double transgenic (*Lrig3*-TG) mice showed sporadic hair loss on their skin. Furthermore, we found a dysregulated expression of fundamental contributors to HF signaling pathways along with perturbed expression levels of multiple HF and SG stem cell markers in the skin of the *Lrig3*-TG mouse line. The present study provides first insights into how LRIG3 contributes to maintenance of skin homeostasis. Moreover, our findings clarify the potential molecular bases behind hair loss in *Lrig3*-TG mice.

## Results

### Mouse generation

**Fig. 1:**
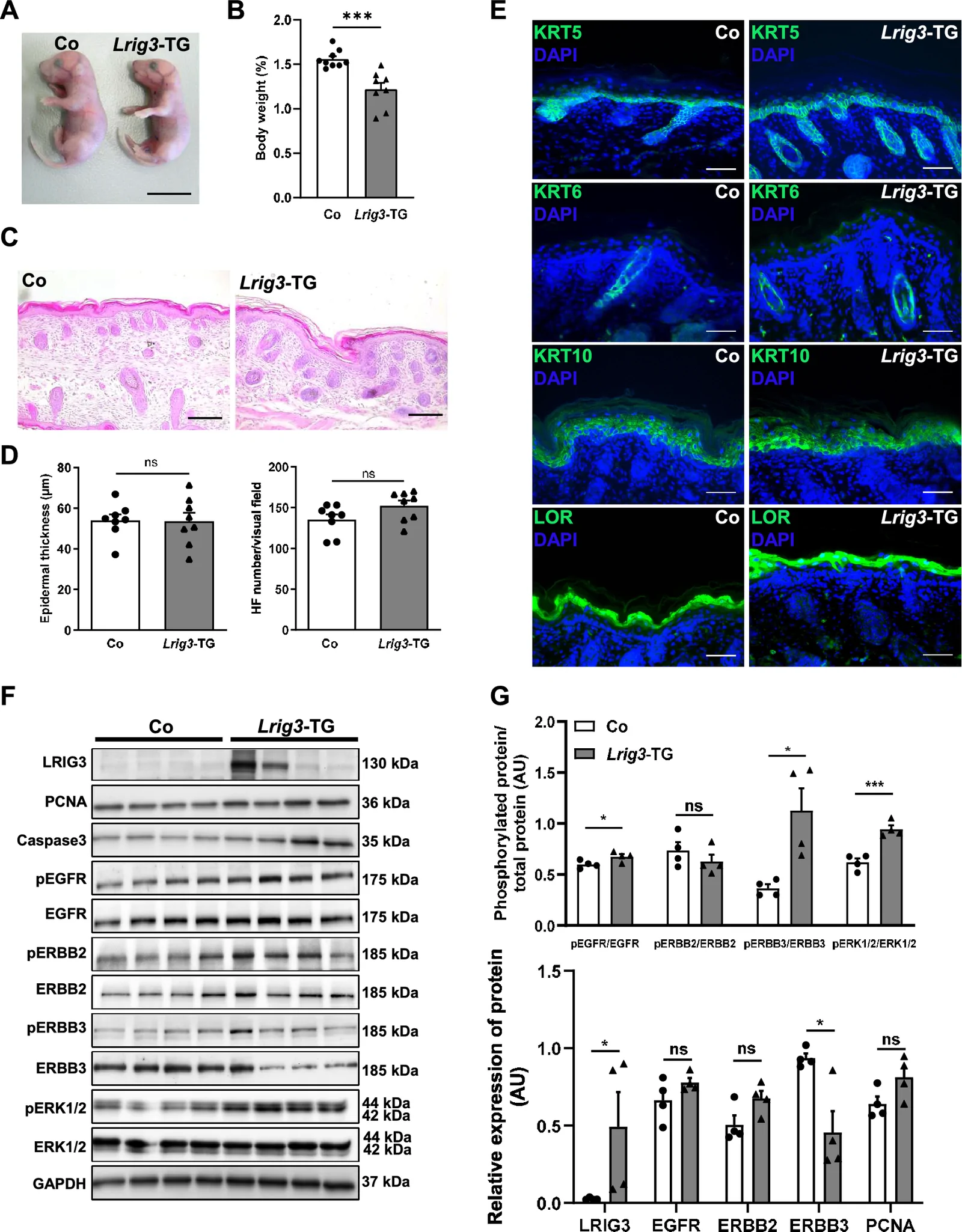
Characterization of LRIG3 overexpression in neonates. (**A**) Representative image of control and *Lrig3*-TG mice at P0. Scale bar indicates 1 cm. (**B**) *Lrig3*-TG newborns markedly exhibited lower body weight compared with controls at P0. 8 *Lrig3*-TG/9 control neonates. (**C**) Histological and (**D**) morphometrical analysis of epidermal thickness and HF number. Scale bars indicate 50 µm. 8 *Lrig3*-TG/9 control neonates. (**E**) Representative IF staining of KRT5 (green), KRT6 (green), KRT10 (green), and LOR (green), on the FFPE skin sections of *Lrig3*-TG and control neonates. Cell nuclei are stained with DAPI (blue). Scale bars represent 50 µm. (**F**) Western blot and (**G**) densitometric analyses on the skin samples of 4 *Lrig3*-TG pups and 4 controls at P0. GAPDH was used as reference protein. (**B**, **D**, **G**) Data were analyzed with Student’s *t*-test. Data are expressed as mean ± SEM (*P < 0.05, **P < 0.01, ***P < 0.001). Unprocessed original western blot images are available in Fig. S1.

To determine the biological consequences of LRIG3 expression in the skin, two independent mouse lines overexpressing LRIG3 in a skin-specific manner were established by the inducible and reversible Tet-Off system. Double transgenic mice were generated by crossing mice expressing tetracycline transactivator under the control of a *Krt5* promoter (*Krt5*-tTA)^25^ with the *Lrig3* Tet-response (pTRE*-Lrig3*) mouse line. Even after numerous breeding, no double transgenic (*Krt5-*tTA/pTRE-*Lrig3* or *Lrig3*-TG) pups were genotyped, suggesting that the overexpression of LRIG3 might lead to lethality during embryonic development. Nevertheless, the analysis of pups immediately after birth revealed that the number of double transgenic embryos followed Mendelian ratios. A toluidine blue staining of the embryos at day E16.5, E17.5, E18.5, and at postnatal day zero (P0) demonstrated no alterations in the skin barrier of transgenic and control animals (data not shown). Analysis of the *Lrig3*-TG pups at P0 showed that they have significantly lower body weight compared with control littermates (Fig. 1A,B) and histological analysis of the skin indicated no marked changes in epidermal thickness or HF number (Fig. 1C,D). The keratinocyte differentiation markers keratin 5 (KRT5)^26^, KRT6^27^, KRT10^28^, and loricrin (LOR)^29^ detected by immunofluorescence (IF) staining were expressed in a similar manner in *Lrig3*-TG and control pups (Fig. 1E), which served as further evidence that epidermal stratification and differentiation were maintained in the skin of the *Lrig3*-TG neonates. However, western blot analysis of skin lysates revealed distinct molecular alterations in the *Lrig3*-TG pups. We observed a reduced expression of ERBB3 and increased activation ratios of pEGFR/EGFR, pERBB3/ERBB3, and pERK1/2 to ERK1/2 (Fig. 1F,G). These findings imply that even though LRIG3 overexpression in the skin does not have a profound effect on overall skin structure, it alters the signaling pathways underlying the maintenance and growth of the skin.

### *Lrig3*-TG mice showed alopecia and an increased epidermal thickness

**Fig. 2:**
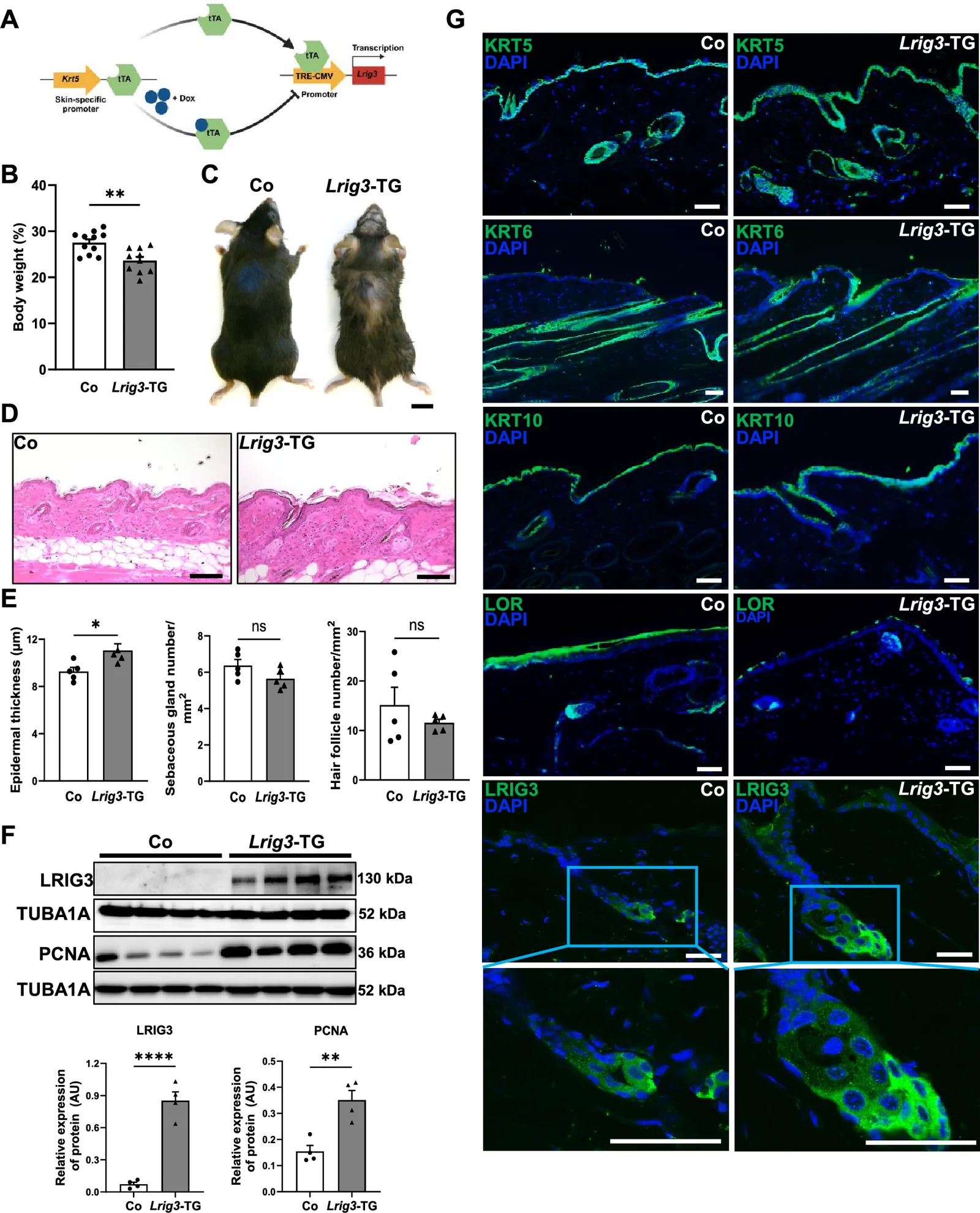
Generation and characterization of *Lrig3*-TG mice. (**A**) *Lrig3*-TG viability management strategy. Using the Tet-Off system, prenatal *Lrig3* overexpression is silenced by administration of dox and following dox withdrawal, continuous *Lrig3* overexpression occurs in the mice after birth. (**B**) *Lrig3*-TG mice gain less weight and are approximately 14% lighter than controls. 10 *Lrig3*-TG/11 control female and male mice at the age of 68 weeks. (**C**) Representative images of skin manifestation from 6-month-old *Lrig3*-TG and control mice. *Lrig3*-TG mouse line shows patchy hair loss on the trunk and head. Scale bar represents 1 cm. (**D**) Histological examination of the skin and (**E**) morphometric analysis revealed thicker epidermis in *Lrig3*-TG mice. 5 *Lrig3*-TG/5 control female mice at the age of 68 weeks. Scale bars denote 50 µm. (**F**) Western blot and densitometric analyses confirmed higher expression of LRIG3 and PCNA proteins in the skin of *Lrig3*-TG mice. TUBA1A was used as an internal loading control. 4 *Lrig3*-TG/4 controls of 68-week-old female mice. Unprocessed original Western blot images are available in Fig. S2. (**G**) Representative images of IF staining of KRT5 (green), KRT6 (green), KRT10 (green), LOR (green), and LRIG3 (green) on the FFPE skin sections of *Lrig3*-TG and control mice. Cell nuclei are stained with DAPI (blue). Scale bars indicate 50 µm. (**B**, **E**, **F**) Data were analyzed with Student’s *t*-test. Data are expressed as mean ± SEM (*P < 0.05, **P < 0.01, ***P < 0.001, ***P < 0.0001).

**Fig. 3:**
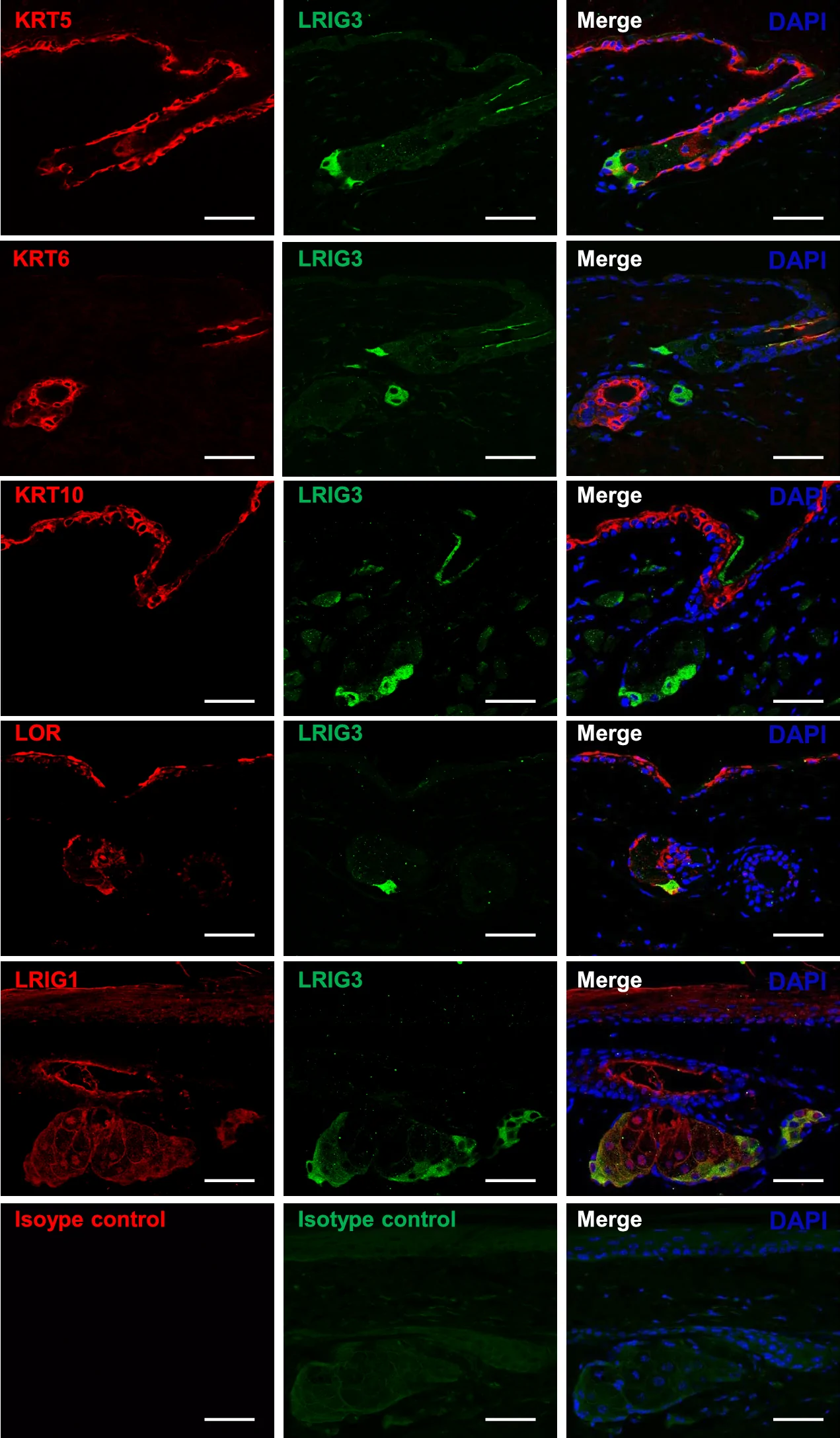
Determination of LRIG3 localization in murine skin. Representative IF staining of KRT5 (red), KRT6 (red), KRT10 (red), LOR (red), and LRIG1 (red) co-stained with LRIG3 (green) on the FFPE skin sections of wild type mice at the age of 6 months. Cell nuclei are stained with DAPI (blue). Scale bars represent 50 µm.

The embryonic LRIG3 expression was suppressed through doxycycline administration. After birth, skin-specific LRIG3 overexpression was induced by withdrawing doxycycline from drinking water, resulting in mice with targeted overexpression of LRIG3 in the skin (Fig. 2A). Akin to the P0 pups, six-month-old *Lrig3*-TG mice showed significantly decreased body weight compared with control littermates (Fig. 2B). The skin of *Lrig3*-TG mice was characterized by intermittent hair loss (Fig. 2C). Histological examination of skin sections revealed no apparent changes in the number of HFs or SGs, but the epidermis was notably thicker in the *Lrig3*-TG mice compared with control mice (Fig. 2D,E). This was supported by an elevated expression of proliferating cell nuclear antigen (PCNA), a well-established indicator of cellular proliferation^30^, in the skin of *Lrig3*-TG mice (Fig. 2F). Moreover, IF analysis of the KRT5^26^, KRT6^27^, and KRT10^28^ demonstrated an unchanged expression pattern of these proteins in both groups (Fig. 2G). While LOR, a key structural component of the epidermal barrier^31^, showed a lower expression in the skin of *Lrig3*-TG mice in comparison with control mice (Fig. 2G). Additionally, to determine LRIG3 localization in the epidermal sub-compartments in wild type mouse skin, the differentiation markers, KRT5, KRT6, KRT10, LOR, and LRIG1, were chosen for their double IF staining with LRIG3. IF results revealed a strong expression of LRIG3 in SGs. Furthermore, we found some positive signals of LRIG3 overlapped with those of KRT6 in the infundibulum of the HF and with LRIG1 in SGs. (Fig. 3).

### Disrupted keratinocyte differentiation and impaired cytoskeletal organization in *Lrig3*-TG mice

**Fig. 4:**
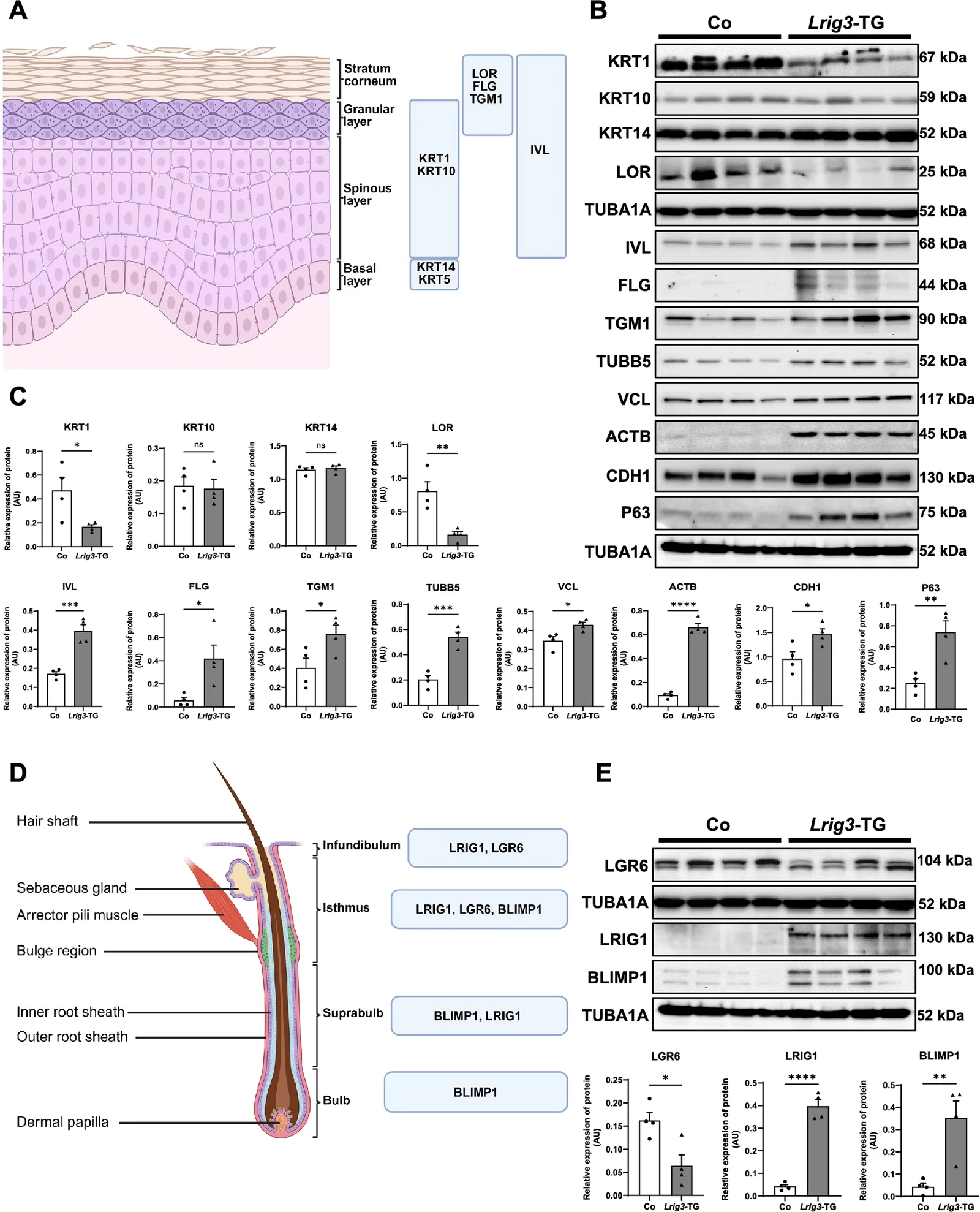
LRIG3 overexpression alters protein expression profile of the epidermis and its appendages. (**A**) Diagram of expression pattern of epidermal differentiation biomarkers. (**B**) Western blots and (**C**) densitometric analyses of various proteins associated with different layers of the epidermis. Western blots quantification was normalized with TUBA1A signal. (**D**) Schematic illustration of the HF depicting compartments of HF and SG stem cell markers. (**E**) Western blot and densitometric analyses showed a perturbed expression of the HF and SG stem cell markers in the back skin of *Lrig3*-TG mice in comparison with control. Densitometric values were normalized with TUBA1A. 4 *Lrig3*-TG/4 controls of 68 weeks old females. Unprocessed original Western blot images are available in Fig. S3. Data were analyzed with Student’s *t*-test and are expressed as mean ± SEM (*P < 0.05, **P < 0.01, ***P < 0.001, ****P < 0.0001).

A detailed investigation of the effects of *Lrig3* overexpression on the epidermis was conducted by analyzing various protein expression profiles across different epidermal layers (Fig. 4A). Western blot analysis was used to examine the expression of a set of proteins contributed to the keratinization, epidermal integrity, keratinocyte differentiation, cornified envelope formation, cytoskeleton structure, and cell adhesion. The results revealed that the expression of KRT1 and LOR was significantly decreased in *Lrig3-*TG mice compared with control littermates. On the contrary the expression of involucrine (IVL), filaggrin (FLG), transglutaminase-1 (TGM1), beta-tubulin (TUBB5), vinculin (VCL), beta-actin (ACTB), and E-cadherin (CDH1) was notably upregulated in the skin of *Lrig3-*TG mice relative to control (Fig. 4B,C). These findings indicate that LRIG3 overexpression has prominently influenced the differentiation of keratinocytes in the suprabasal layer of the epidermis (spinous and granular layers) and a compensatory response to the reduction of KRT1 and LOR, leads to the disturbed differentiation process in the skin of *Lrig3*-TG mice. More interestingly, we detected higher expression of P63 in the skin of *Lrig3*-TG mice when compared with controls (Fig. 4B,C). In addition to its established role in keratinocyte differentiation and proliferation, P63 is crucial for HF development and maintenance, with a dominant expression in the bulge and bulb regions^32–34^. Its upregulation in the skin of the *Lrig3*-TG mice may be responsible for an imbalanced HF homeostasis, which potentially explains the hair loss manifestation in this mouse line.

### Hair follicle stem cell niche is disrupted in *Lrig3*-TG mice

Stem cells represent the core source of the building blocks for sustaining tissue homeostasis and facilitating its reconstruction^35^. To interrogate the contribution of the *Lrig3* gene to HF and SG development and growth, we examined the expression levels of proteins linked to the HF and SG stem cells (HFSCs), including BLIMP1, LRIG1, and LGR6 (Fig. 4D) in the skin of *Lrig3*-TG and control mice. The expression of BLIMP1 is associated with the dermal papilla during hair morphogenesis and throughout the postnatal hair cycle^36^ and SGs^37^. LRIG1 is predominantly expressed in the junctional zone (JZ)^15^ and isthmus of the HF^38^ and development of SGs^15^. LGR6, a transmembrane protein, is mostly expressed in the isthmus area^39^ and it contributes to generation of SGs^40^. These proteins all work together under meticulous control to coordinate the HF and SG development. Using western blot analysis, we detected a reduced expression of LGR6 coupled with an elevated expression of BLIMP1 and LRIG1 in the skin of the *Lrig3*-TG mice when compared with control mice (Fig. 4E). The altered expression of BLIMP1, LRIG1, and LGR6 proteins in the skin of *Lrig3*-TG mice points to a potential imbalance between stem cell quiescence and proliferation. This may provide an initial explanation for the hair loss phenotype in the *Lrig3*-TG mouse line.

### ERBB and NOTCH signaling pathways are dysregulated in *Lrig3*-TG mice

**Fig. 5:**
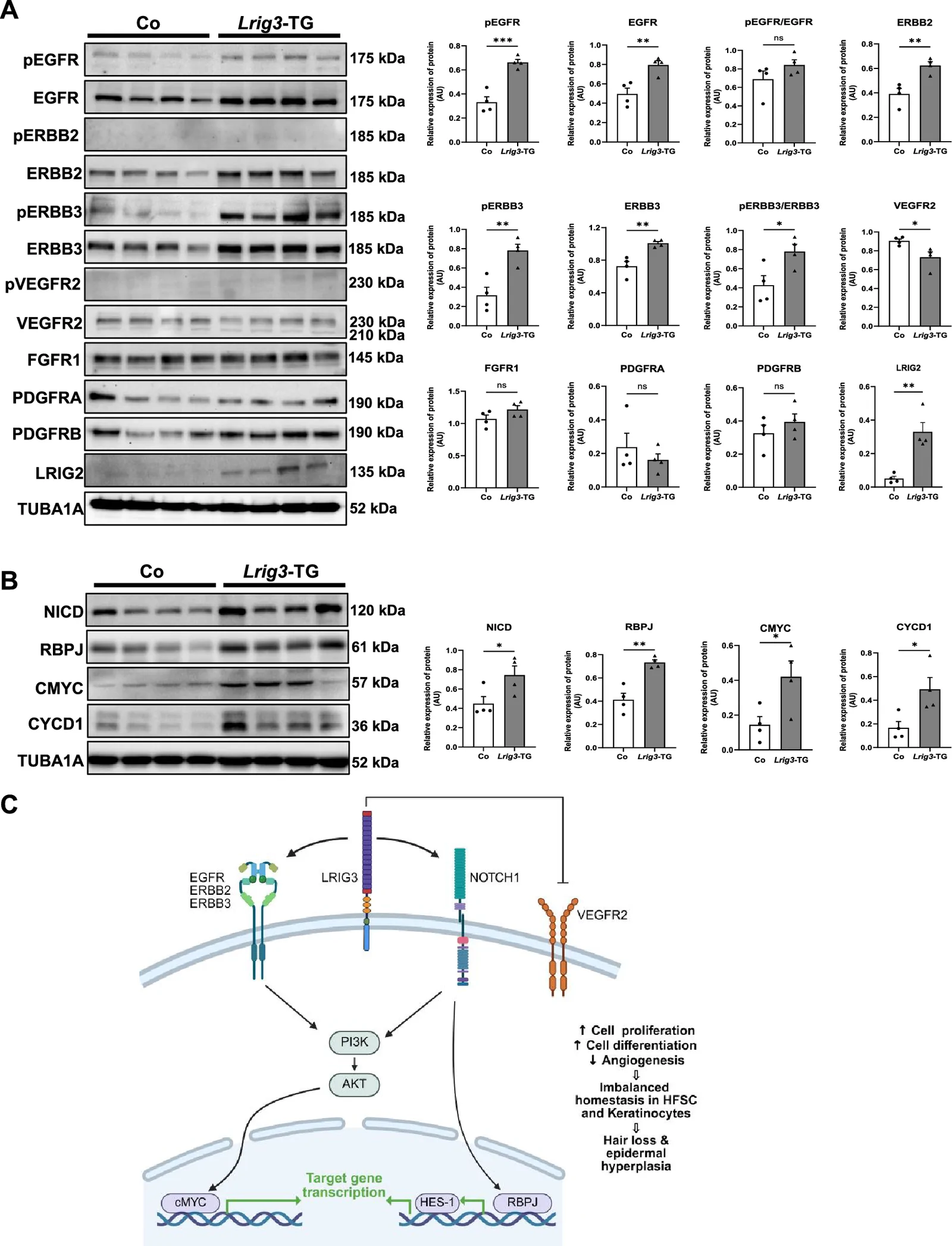
HF-associated signaling pathways are dysregulated in the skin of *Lrig3*-TG mice. (**A**) Western blot and densitometric evaluations were performed in back skin of the *Lrig3*-TG and control mice to assess the expression of ERBB and various growth factor receptors, potential targets of LRIG3 that play crucial role in HF signaling. (**B**) Western blot and densitometric analyses demonstrate the expression level of various NOTCH signaling components, a key pathway in HF signaling, in the skin of *Lrig3*-TG and control mice. TUBA1A protein served as an internal control for normalizing the densitometric values. 4 *Lrig3*-TG/4 controls of 68 weeks old females. Unprocessed original western blot images are available in Fig. S4. The Student’s *t*-test was used for data analysis, and values are represented as mean ± SEM (*P < 0.05, **P < 0.01, **P < 0.001). (**C**) A hypothetical mechanism of action of LRIG3 in the skin of *Lrig3*-TG mice leading to hair loss or alopecia. LRIG3 overexpression in the skin induces the stabilization and activation of ERBB receptors, including EGFR, ERBB2, and ERBB3 as well as stimulation of the NOTCH signaling pathway. PI3K/AKT acts as a common downstream mediator linking NOTCH and ERBB receptor signaling, leading to the activation of transcription factors such as CMYC. Concurrently, NOTCH cascade triggers the activation of RBPJ and CMYC. RBPJ drives transcriptional activation of multiple target genes such as HES-1 involved in cell proliferation and differentiation. In contrast, LRIG3 suppresses expression of VEGFR2 protein, a contributor to HF proliferation and differentiation. The imbalanced molecular cascades in the skin of *Lrig3*-TG mice affect HF function, which ultimately disrupt HF homeostasis and result in hair loss or alopecia.

**Fig. 6:**
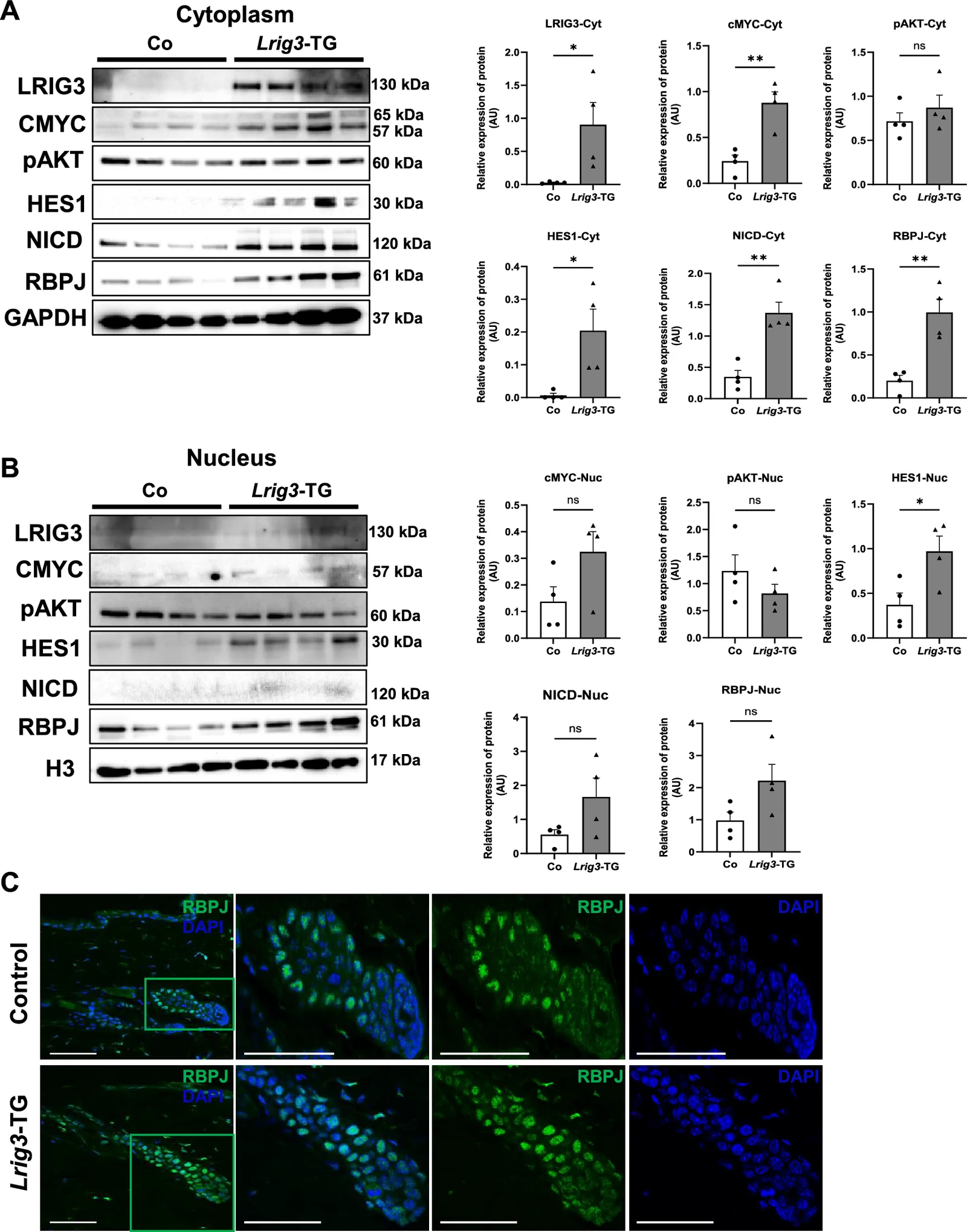
Cytoplasmic and nuclear fractions exhibit evidence of activated downstream NOTCH signaling. (**A**) Western blot and densitometric analyses of cytoplasmic fraction on skin samples of 4 *Lrig3*-TG animals and 4 controls at the age of 68 weeks. GAPDH was used as reference protein. (**B**) Western blot and densitometric analyses of nuclear fraction on skin samples of 4 *Lrig3*-TG animals and 4 controls at the age of 68 weeks. Histon 3 (H3) was used as reference protein. Data were analyzed with Student’s *t*-test. Data are expressed as mean ± SEM (*P < 0.05, **P < 0.01). Unprocessed original western blot images are available in Fig. S5. (**C**) Nuclear IF staining of RBPJ (green) in the FFPE back skin sections of *Lrig3*-TG and control mice. Cell nuclei are stained with DAPI (blue). Scale bars indicate 50 µm.

Given the crucial role of LRIG proteins in modulating growth factor receptors as the eminent players in HF development, we deliberated the expression levels of a range of growth factor receptors such as EGFR, ERBB2, ERBB3^3^, FGFR^41^, platelet-derived growth factor receptor alpha (PDGFRA)^42^, PDGFRB^43^, and vascular endothelial growth factor receptor 2 (VEGFR2) in the skin of *Lrig3*-TG and control mice. In relation to the control group, *Lrig3*-TG mice showed an increased expression of EGFR, ERBB2, ERBB3, and their phosphorylated forms, pEGFR and pERBB3, in the skin, suggesting a potential regulatory role of LRIG3 in stabilizing these receptors (Fig. 5A). Further investigation into the HF-related signaling cascades revealed that there is an elevated expression of NOTCH intracellular domain (NICD) in the total lysate and cytoplasmic fractions but not in nuclear fractions of the skin in *Lrig3*-TG mice compared with the control mice (Figs. 5B and 6A,B). In addition, expression of recombination signal binding protein for immunoglobulin J region (RBPJ) protein, a principal transcription factor downstream of NOTCH, in the total lysate and cytoplasmic fraction of *Lrig3*-TG mice’s skin was upregulated (Fig. 5B, Fig. 6A), which was confirmed by IF nuclear staining (Fig. 6C) indicating activation of the NOTCH pathway (Fig. 5B). Moreover, the expression of Hairy and Enhancer of Split-1 (HES1), an essential effector of the NOTCH signaling pathway^44^, downstream of the RBPJ, showed higher expression in both cytoplasmic and nuclear fractions of the skin of the *Lrig3*-TG mice when compared with control group (Fig. 6A,B). The known crosstalk between NOTCH1 and ERBB receptors may account for the observed results^45^. Moreover, we detected a rise in the expression of CYCD1 and CMYC (Fig. 5B), two well-established targets of NOTCH signaling^46,47^, in the skin of *Lrig3*-TG mice in comparison with controls. Likewise, an enhanced expression of CMYC was observed only in the cytoplasmic fraction of the skin in *Lrig3*-TG compared with the wild type controls (Fig. 6A). A lower expression of VEGFR2 was also detected in the back skin of *Lrig3*-TG mice compared with controls (Fig. 5A). All in all, the overexpression of LRIG3 alters key pathways essential for HF development, such as activation of EGFR and NOTCH signaling pathways. Consequently, increased levels of CMYC and CYCD1 lead to epidermal hyperplasia. The output of several disturbed signaling pathways may hamper HF development and ultimately result in hair loss in the *Lrig3*-TG mice.

### LRIG3 signaling is mediated by PI3K/AKT pathway

**Fig. 7:**
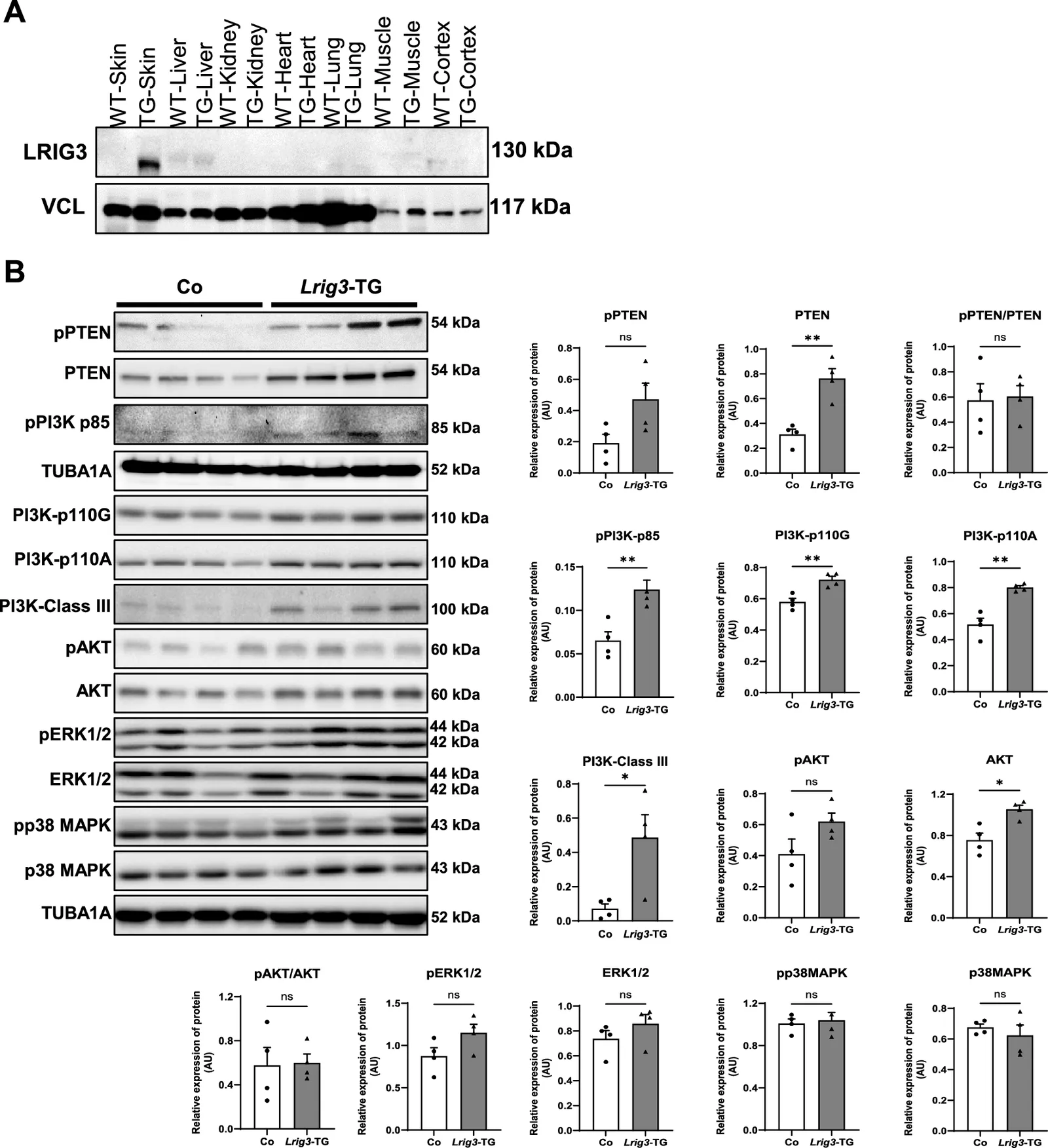
Analysis of transmitting signaling pathways in the skin of *Lrig3*-TG mice. (**A**) Western blot analysis of LRIG3 in various organs of adult *Lrig3*-TG and control mice at the age of 68-week-old verified the skin-specific overexpression of LRIG3 in the *Lrig3*-TG mice. VCL was used as an internal loading control. (**B**) PI3K/AKT, an essential signaling cascade in HF regeneration, is activated in the skin of *Lrig3*-TG mice. Western blot and densitometric analyses were used to assess the protein expression of the constituents of different signal transducing pathways in the back skin of the *Lrig3*-TG and control mice. Normalization of densitometric values was performed with TUBA1A. 4 *Lrig3*-TG/4 controls of 68 weeks old females. Unprocessed original western blot images are available in Fig. S6. For data analysis, the Student’s *t*-test was used and values are expressed as mean ± SEM (*P < 0.05, **P < 0.01).

To dissect the foundational transmitting signals triggered by the activated ERBB and NOTCH receptors, we evaluated the expression profile of multiple contributors to the corresponding signaling pathways in the skin of the *Lrig3*-TG and control mice using western blot analysis. From the PI3K/AKT pathway, we examined the expression of Class III PI3K, PI3K p110A, and PI3K p110G, as well as AKT. From the mitogen-activated protein kinase (MAPK)/extracellular signal-regulated kinase (ERK) pathway, we analyzed the expression levels of ERK1/2 and p38MAPK. No changes were observed in the expression of ERK1/2 and p38MAPK as well as in their phosphorylated forms between *Lrig3*-TG and control mice (Fig. 7B). A marked increase in the expression of Class III PI3K, PI3K p110A, PI3K p110G, and the regulatory subunit of PI3K, PI3K p85, was detected in *Lrig3*-TG mice compared with control littermates (Fig. 7B). An enhanced expression level of AKT was also observed in the skin of *Lrig3*-TG in comparison with control mice (Fig. 7B). This result is corroborated by our earlier findings in this mouse line, which demonstrated an upregulation of CMYC and CYCD1, two downstream effectors within the PI3K/AKT pathway^48,49^, in the skin of *Lrig3*-TG compared with that of control mice. Moreover, the activation of the PI3K/AKT cascade by both the EGFR^49,50^ and NOTCH^51^ pathways are previously recognized. This allows us to conceptualize a signaling mechanism that illustrates the interplay between these two pathways activated in the skin of *Lrig3*-TG mice (Fig. 5C). To our surprise, a higher expression of PTEN was also detected in the skin of *Lrig3*-TG compared with control mice (Fig. 7B). This can be explained by a negative feedback loop in which the activation of the PI3K/AKT pathway stimulates expression and activation of PTEN to keep the PI3K/AKT pathway in check, preventing its overactivation^52^ and sustaining the tissue homeostasis.

## Discussion

LRIG3 diversely functions in the tissues through distinct signaling pathways. It modulates RET receptor signaling in sensory neurons in the nervous system^23^. In Xenopus, LRIG3 is also essential for neural crest induction, acting through modulation of Wnt and FGF signaling cascades^22^. LRIG3 is required for inner ear morphogenesis and craniofacial formation throughout embryonic development^21^. In the context of tumorigenesis, by modulating cell proliferation and angiogenesis, LRIG3 acts as a tumor suppressor in a variety of cancers^53,54^. The multifaceted roles of LRIG3 underscore its importance in preserving tissue homeostasis and development. The impact of LRIG3 on skin development and function is yet to be understood. Here, for the first time, we investigated the consequences of LRIG3 overexpression on the function and architectural integrity of the skin. The visible hair loss was the most prominent effect on the skin of double transgenic (*Lrig3*-TG) mice, which started at 12 weeks of age. Apart from apparent epidermal hyperplasia in the skin of *Lrig3*-TG mice, there were no overall histological changes in the skin of *Lrig3*-TG mice. Previous studies have reported that among transgenic mice overexpressing various LRIG family proteins, the *Lrig1*-TG mouse line demonstrates alopecia, impaired HF morphogenesis and cycling, along with epidermal hyperplasia^19^. While no noticeable abnormalities in the HF and epidermis have been detected in the skin of the *Lrig2*-TG mice^20^. At the molecular level, when studying the key proteins linked to skin differentiation and proliferation, we detected profound alterations in the skin of *Lrig3*-TG mice. Epidermal hyperproliferation disrupts normal differentiation patterns in keratinocytes^55^. In our mouse model, hyperproliferative epidermis was demonstrated by epidermal hyperplasia and increased expression of CYCD1, CMYC, and PCNA. We also observed reduced levels of the early differentiation marker, KRT1, alongside elevated levels of multiple late differentiation markers IVL, FLG, and TGM1, as well as upregulation of skin structural proteins such as VCL, TUBB5, ACTB, and CDH1. These findings suggest an impaired transition from early to late keratinocyte differentiation. To compensate for this defect and maintain skin integrity and barrier function, late differentiation markers are upregulated. Altered expression pattern of keratinocyte differentiation markers in the skin of *Lrig1*-TG mice further supports our findings^19^. Moreover, two factors prompted us to examine the expression of several HF and SG stem cell proteins in the skin of *Lrig3*-TG mice: the prominent expression of LRIG3 in SGs and infundibulum of the HF, and detection of differential expression of P63 protein in the skin of these mice, as a master gene for the development of skin and its derivatives, particularly HF^33^. The imbalance of the stem cell niche within the HF and SG in the skin of *Lrig3*-TG mice was indicated by upregulation of BLIMP1 and LRIG1 and downregulation of LGR6 which may result in disrupted HF growth. Increased expression of transcripts of the HF and SG stem cell markers, *Blimp1*, *Cd34,* and *Lhx2,* has been reported in the skin of *Lrig1*-TG mice^19^. Within the LRIG protein family, LRIG1 is a well-characterized stem cell marker known as a regulator of stem cell quiescence within diverse tissues, including the epidermis, the hard palate of the oral mucosa, the intestinal epithelium, the brain, and the incisors^56^. Likewise, LRIG3 has been found to be a regulator of stem cells in the intestine by modulating stem cell compartment size and preserving the structural integrity of colonic crypts^57^. A strong hypothesis suggests that LRIG1 and LRIG3 modulate stem cells, mainly in quiescence and signaling^56^. Furthermore, we showed that LRIG3 promotes the expression of EGFR, ERBB2, and ERBB3 with concurrent activation of EGFR and ERBB3, highlighting its role as a positive regulator of ERBB signaling. This result is in agreement with the stimulatory role of LRIG2 in ERBB signaling reported in *Lrig2*-TG mice^20^. Conversely, LRIG1 functions as a negative modulator of ERBB signaling in the skin of *Lrig1*-TG mice^19^, reinforcing its well-established inhibitory role in ERBB signaling^1^. The previously reported opposing impact of LRIG3 on LRIG1 activity^58^ further corroborates the observed enhancing effect of LRIG3 on the ERBB signaling in our mouse line. This may account for the elevated expression of LRIG1 in the skin of the *Lrig3*-TG mice via a suppressive feedback mechanism. In this scenario, the activated ERBB pathway triggered by overexpression of LRIG3 leads to a rise in LRIG1 expression to mitigate the signaling. We also detected an enhanced activation of NOTCH signaling (increased expression of NICD and its transcription factor, RBPJ, as well as its essential effector, HES-1) in the skin of the *Lrig3*-TG mice. It has previously been demonstrated that NOTCH signaling through *Ntn1*, a NOTCH signaling component, interacts with LRIG3, and plays a role in shaping the inner ear structure^21^. The NOTCH-LRIG signaling crosstalk has also been reported in *Lrig1*-TG^19^ and *Lrig2*-TG^20^ mice. To confirm direct physical interaction between LRGI3 and ERBB receptors as well as NOTCH, additional co-immunoprecipitation analysis is warranted. While our data demonstrates that overexpressing LRIG3 leads to significant alterations in ERBB and NOTCH-associated signaling in the skin of *Lrig3*-TG mice, the specific epidermal cell populations contribute to these changes have not yet been fully characterized. Although the main conclusions of the current study are reinforced by the consistent histological and molecular alterations, we acknowledge that single-cell transcriptomic (scRNA-seq) analysis would be the ideal next step to further clarify whether hair loss phenotype is driven by a primary defect in the stem cell niche or a secondary destruction in keratinocyte differentiation. Moreover, our data indicate that ERBB and NOTCH signaling is transduced through the PI3K/AKT pathway, evidenced by enhanced expression of Class III PI3K, PI3K p110A, PI3K p110G, PI3K p85, and AKT as well as its downstream targets such as CYCD1 and CMYC in the skin of *Lrig3*-TG mice. Similarly, in *Lrig1*-TG and *Lrig2*-TG mice, findings consistent with ours were observed, with the PI3K pathway being the mediator through which LRIGs exert their roles^19,20^. Contrary to our results, the study on human glioma cell lines identified that LRIG3 mediates its function by suppressing the PI3K/AKT pathway^54^. A skin-specific LRIG3 knockout mouse model would have enabled a more precise investigation of the physiological function of LRIG3 in the skin. Although in the current study, we analyzed a skin-specific transgenic LRIG3 mouse model, the observed phenotype is driven by its ectopic overexpression and therefore likely reflects conditions primarily associated with pathological settings, including alopecia-related phenotypes. Nevertheless, the finding that LRIG3 overexpression induces alopecia is of considerable interest, particularly since a comparable phenotype has also been reported following loss of LRIG1. Collectively, these observations support the concept that LRIG3 and LRIG1 exert functionally opposing roles in the skin while both contributing to the maintenance of hair follicle homeostasis. Given the minimal physiological expression of LRGI3 in the skin, utilization of *Lrig3*-KO mouse line would lead to more robust conclusion that the observed phenotype and signaling alterations are driven specifically by LRIG3 overexpression in *Lrig3*-TG mice.

In conclusion, the hair loss phenotype in our *Lrig3*-TG mouse line likely involves the intricate interplay of several signaling pathways. Overexpression of LRIG3 in the skin is associated with a cascade of events, including upregulation and activation of ERBB and NOTCH signaling potentially through the PI3K/AKT pathway, coupled with downstream upregulation of CMYC and CYCD1; however, it appears to suppress the expression of VEGFR2. These molecular changes correlate with abnormal epidermal proliferation and differentiation, as well as disruption of HFSC niches, which together may contribute to hair loss (Fig. 5C). *Lrig3*-TG mice demonstrate several phenotypic and molecular features that align with known mechanisms underlying alopecia. These findings suggest the potential of the *Lrig3*-TG mouse line as a model for studying HF biology and could provide new insights into therapeutic intervention in hair loss conditions.

## Materials and methods

### Ethical regulations

The animal study was reviewed and approved by the Ethics and Welfare Committee of the University of Veterinary Medicine Vienna (approval number: 2021–0.878.127). All animal experiments were conducted in accordance with the requirements of the national authority (Austrian Federal Ministry of Education, Science, and Research) and complied with §§ 26ff. of the Austrian Animal Experiments Act (Tierversuchsgesetz 2012 – TVG 2012) under license numbers BMBWF-68.205/0010-V/3b/2019 and 2021–0.878.127. We confirm that all experiments were performed in accordance with relevant guidelines and regulations of the Ethics and Welfare Committee of the University of Veterinary Medicine, Vienna, and the Austrian Federal Ministry of Education, Science, and Research. In brief, for this study we used skin from adult and newborn mice. The animals were humanely euthanized, adult mice were euthanized by cervical dislocation, and the newborn mice were decapitated using scissors. Further details regarding the animal experiments are provided in the section ‘Mouse generation and experiments’. Furthermore, qualified personnel executed all animal experiments. The authors took the ARRIVE guidelines into account when preparing the manuscript and applied them at the appropriate sections.

### Mouse generation and experiments

Pronuclear microinjection into zygotes of C57BL/6N mice was performed to generate two independent mouse lines that overexpress LRIG3 specifically in the skin. The zygotes were transferred into the oviduct of recipient mice. All recipients received for anesthesia an intraperitoneal injection of ketamine and xylazine (Ketasol, Xylasol, both Graeub, Switzerland; 10 mg/100 g body weight (bw) ketamine; 0.4 mg/100 g bw xylazine; 0.07 ml per 10 g bw). Once the necessary anesthetic depth was reached, the eyes were covered with eye ointment (Oleovit, Fresenius Kabi, Austria). For analgesia, 0.05 mg/100 g bw meloxicam (Metacam; Boehringer Ingelheim, Germany) was given subcutaneously once before surgery. To achieve the new transgenic mouse lines, *Lrig3* cDNA was cloned into the pTRE-tight vector (Takara, Saint-Germain-en-Laye, France). Two pTRE-tight-*Lrig3*-TG mouse lines were generated and they were mated with the previously described keratin 5 (*Krt5*) promoter driver mouse line (*Krt5*-tTA), expressing the tetracycline-regulated transcriptional transactivator (tTA) under the control of the *Krt5* promoter^25^. This resulted in two independent *Lrig3*-TG mouse lines that express exogenous LRIG3 specifically in the skin. Both mouse lines exhibited the same phenotype, all experiments presented in this manuscript were conducted using one line, referred to as *Lrig3*-TG (*Lrig3*-TG: TRE-*Lrig3*;*Krt5*-tTA (C57BL/6N-Tg(pTRE-Lrig3)-Tg(K5-tTA)), controls (Co): wildtype (C57BL/6N), *Krt5*-tTA (C57BL/6N-Tg(K5-tTA), TRE-*Lrig3 (C57BL/6N-Tg(pTRE-Lrig3)*). C57BL/6N mice were purchased form the commercial breeder Janvier (France). All mouse strains were maintained in the C57BL/6N background and were bred and housed in specific pathogen-free conditions in the mouse facility of the University of Veterinary Medicine in Vienna. All mice were kept under standard housing conditions (21 ± 1 °C room temperature, 50 ± 10% humidity, 12:12 h light–dark cycle with lights on at 6:00 a.m.) in IVC type IIL mouse cages (Tecniplast, Italy) equipped with wood bedding (Lignocel Select, J. Rettenmaier und Söhne GmbH + Co-KG, Austria), nesting material (Pur-Zellin® tissue swabs, Paul Hartmann GmbH, Austria) and houses or tubes (SDS Deutschland c/o Jung GmbH, Germany) as enrichment. Commercial mouse diet (V1534, Ssniff GmbH, Germany) and water (chlorinated tap water, 3–4 ppm) were offered *ad libitum*.

For tissue collection, the adult mice were euthanized by cervical dislocation, and the newborn mice were decapitated using scissors. Mice were dissected and skin samples were either snap-frozen or embedded in paraffin. Both male and female mice were included in this study and no sex-based differences were observed (P0: sex not known; six months: female; molecular biological analysis was performed using female mice only). To prevent *Lrig3*-TG expression during embryogenesis, all pregnant mice were given 3 mg/mL doxycycline (Dox) (Beladox 500 mg/g, bela-pharm, Lehnecke 793–588, Schortens, Germany) in the drinking water together with 5% sucrose (#S0389, Sigma, Taufkirchen, Germany) until they gave birth. The skin-specific overexpression in this mouse line was confirmed by evaluating the LRIG3 protein expression in various tissues using western blot analysis (Fig. 5A).

### Western blot analysis

To isolate protein from the skin tissues, frozen samples (~ 50 mg) were disrupted in tissue lysis buffer [HEPES (pH 7.3), 150 mM NaCl, 10% glycerol, 1.5 mM MgCl_2_, 1% Triton X-100, 1 mM EDTA] containing proteinase inhibitor (1:100, Merck KGaA, Darmstadt, Germany) and phosphatase inhibitor (1:10, Merck KGaA, Darmstadt, Germany) using MiniBatch D-1 homogenizer (MICCR® GmbH, Baden-Württemberg, Germany). The total protein concentration of the samples was determined using a Pierce™ Detergent-compatible Bradford Assay Kit (ThermoFisher Scientific, Dreiech, Germany). 30 µg of protein samples from the skin tissue was separated by 10% sodium dodecyl sulfate polyacrylamide gel electrophoresis (SDS-PAGE) and subsequently transferred to a polyvinyl difluoride membrane (Millipore, Schwalbach, Germany) by semidry blotting (Trans-Blot Turbo Transfer System) (Bio-Rad Laboratories, Hercules, CA, USA). The membranes were blocked with 5% non-fat dry milk in TBST (200 mM Tris, pH 7.6, 150 mM NaCl, and 0.1% Tween 20) solution. They were then incubated with the primary antibodies (Supplementary Table 1) at 4 °C for 16 h. Afterward, the membranes were reacted with secondary antibodies (Supplementary Table 1) for 1 h at room temperature (RT). Protein detection on the membrane was carried out using the clarity western ECL substrate (Bio-Rad Laboratories GmbH). To analyze the reference proteins and assess both the phosphorylated state and total protein, membranes were stripped with a stripping buffer (2% SDS, 62.5 mM Tris/HCl, pH 6.7 and 100 mM β-mercaptoethanol) for 40 minutes at 60°C. Images were acquired and exported using Image Lab software from BioRad Laboratories and densitometry quantification of specific protein bands was conducted using ImageJ software (https://imagej.net/ij/download.html). The values were normalized to the tubulin alpha 1 (TUBA1A) or glyceraldehyde-3-phosphate dehydrogenase (GAPDH) loading control.

### Hematoxylin and eosin (H&E) examination of skin samples and morphometric analysis

Skin specimens from the back were fixed in a 4% PFA solution, then dehydrated through a series of alcohols, cleared with xylene, and embedded in paraffin. They were sectioned at a 6-μm thickness, deparaffinized, rehydrated, stained with H&E, and finally cover-slipped with a xylene-based mount medium. All sections were visualized, and high-resolution whole slide images were captured using the SLIDEVIEW™ VS200 research slide scanner (Olympus Life Sciences, Waltham, MA, USA). The images were analyzed using OlyVIA software to assess epidermal thickness, HFs, and SGs. Briefly, to evaluate the epidermal thickness, the whole slide image was viewed in OlyVIA software at 400 × magnification. The area of the epidermis was manually outlined using the freehand polygon tab in the toolbox. The length of the given area was measured, and the epidermal thickness was calculated by dividing the epidermal area by its length. The freehand polyline tab was also used to quantify skin appendages like HFs and SGs. By clicking on the object and right-clicking swiftly, a numerical value appears on the selected object, allowing us to count the skin appendages.

### Immunofluorescence analysis of skin samples

For target antigen retrieval, formalin-fixed paraffin-embedded sections were first deparaffinized and then boiled in a microwave for 20 minutes in a 10 mM citrate buffer (pH 6.0). After washing with PBS, non-specific binding of the primary antibodies was blocked by incubating the sections in the blocking buffer, 5% normal donkey serum in 1X TBS, for 1 h at RT. Skin sections were incubated with primary antibodies (Supplementary Table 1) over night at 4 °C, followed by an incubation with secondary antibodies (Supplementary Table 1) for 1 h at RT. After washing with TBS, the slides were mounted with VECTASHIELD® Antifade Mounting Medium with DAPI (Vector Laboratories, CA, USA) and incubated at 4 °C overnight. The sections were then visualized using a Zeiss Axio Imager Z2 microscope or Zeiss LSM 880 Airyscan-confocal microscope (Carl Zeiss, Germany).

### Nuclear fractionation

50 mg of skin samples in hypotonic lysis buffer (10 mM Tris–HCl, pH7.5, 10 mM NaCl, 3 mM MgCl2, 0.3% NP-40, and 10% Glycerol supplemented with protease and phosphatase inhibitors) were homogenized using homogenizer (MICCR® GmbH, Baden-Württemberg, Germany). After 4-minute centrifugation at 250 × g at 4 °C, supernatant, cytoplasmic fraction, was collected. Nuclear pellet was washed 3 times by adding 500 µL of wash buffer (10 mM Tris–HCl, pH7.5, 10 mM NaCl, 3 mM MgCl2, and 10% Glycerol supplemented with protease and phosphatase inhibitors). The nuclear pellet was resuspended in 100 µL of nuclear lysis buffer (250 mM Tris–HCl, pH7.5, 60 mM KCl, and 1 mM EDTA pH8.0 supplemented with protease, phosphatase inhibitors and 25 U/ml benzonase). To mechanically rupture the nuclear envelope, the suspension was exposed to 8 freeze & thaw cycles in liquid nitrogen and 37 °C 2 minutes in each. After centrifugation at 16,000 × g for 5 minutes at 4 °C, supernatant, nuclear fraction protein, was collected. The protein concentration was determined using a Pierce Detergent-compatible Bradford Assay Kit (ThermoFischer Scientific, Dreiech, Germany).

### Statistical data analysis

GraphPad Prism v10 (GraphPad Software, CA, USA) was used for the statistical analysis of the data. Values are presented as the mean ± standard error of the mean (SEM). An unpaired Student’s *t*-test was used to compare the means and determine the *P*-value of two groups. A *P*-value < 0.05 was considered statistically significant. Figures 4A,D and 5C were created with BioRender software (BioRender, Toronto, Canada).

## Supplementary Information


Supplementary Information 1.
Supplementary Information 2.
Supplementary Information 3.
Supplementary Information 4.
Supplementary Information 5.
Supplementary Information 6.
Supplementary Information 7.


## Data Availability

The data that support the findings of this study are available from the corresponding author [maik.dahlhoff@vetmeduni.ac.at] upon reasonable request.

## Abbreviations

AU: Arbitrary unit
BLIMP1: B lymphocyte-induced maturation protein 1
CDH1: E-cadherin
CMYC: Cellular myelocytomatosis oncogene
CYCD1: Cyclin D1
Dox: Doxycycline
EGFR: Epidermal growth factor receptor
ERK: Extracellular signal-regulated kinase
FFPE: Formalin-fixed paraffin-embedded
FGF: Fibroblast growth factor
FLG: Filaggrin
HES-1: Hairy and Enhancer of Split-1
HF: Hair foliclle
HFSC: Hair foliclle stem cell
IF: Immunofluorescence
IVL: Involucrin
KRT: Keratin
LGR6: Leucine-rich repeat-containing G-protein-coupled receptor 6
LOR: Loricrin
LRIG: Leucine-rich repeat and Immunoglobulin-like domains
MAPK: Mitogen-activated protein kinase
PDGFR: Platelet-derived growth factor receptor
RBPJ: Recombination signal binding protein for immunoglobulin J region
RET: Rearranged during transfection
RTK: Receptor tyrosine kinases
SEM: Standard error of the mean
SG: Sebaceous gland
TGM1: Transglutaminase-1
TRE: Tetracycline response element
TUBA1A: Tubulin alpha 1a
TUBB5: Tubulin beta 5 class I
VCL: Vinculin
VEGFR2: Vascular endothelial growth factor receptor 2
Wnt: Wingless-type MMTV integration site family
