# Supplementary material for: Cutaneous gain-of-function mutation of LRIG3 leads to alopecia by upregulation of ERBB, PI3K/AKT, NOTCH1 signaling pathways
Source: Sci Rep. 2026 May 30;16:27334. doi: 10.1038/s41598-026-54295-5 (PMC13530143; doi:10.1038/s41598-026-54295-5)
Supplement: Supplementary file 1 [file 41598_2026_54295_MOESM1_ESM.pdf]

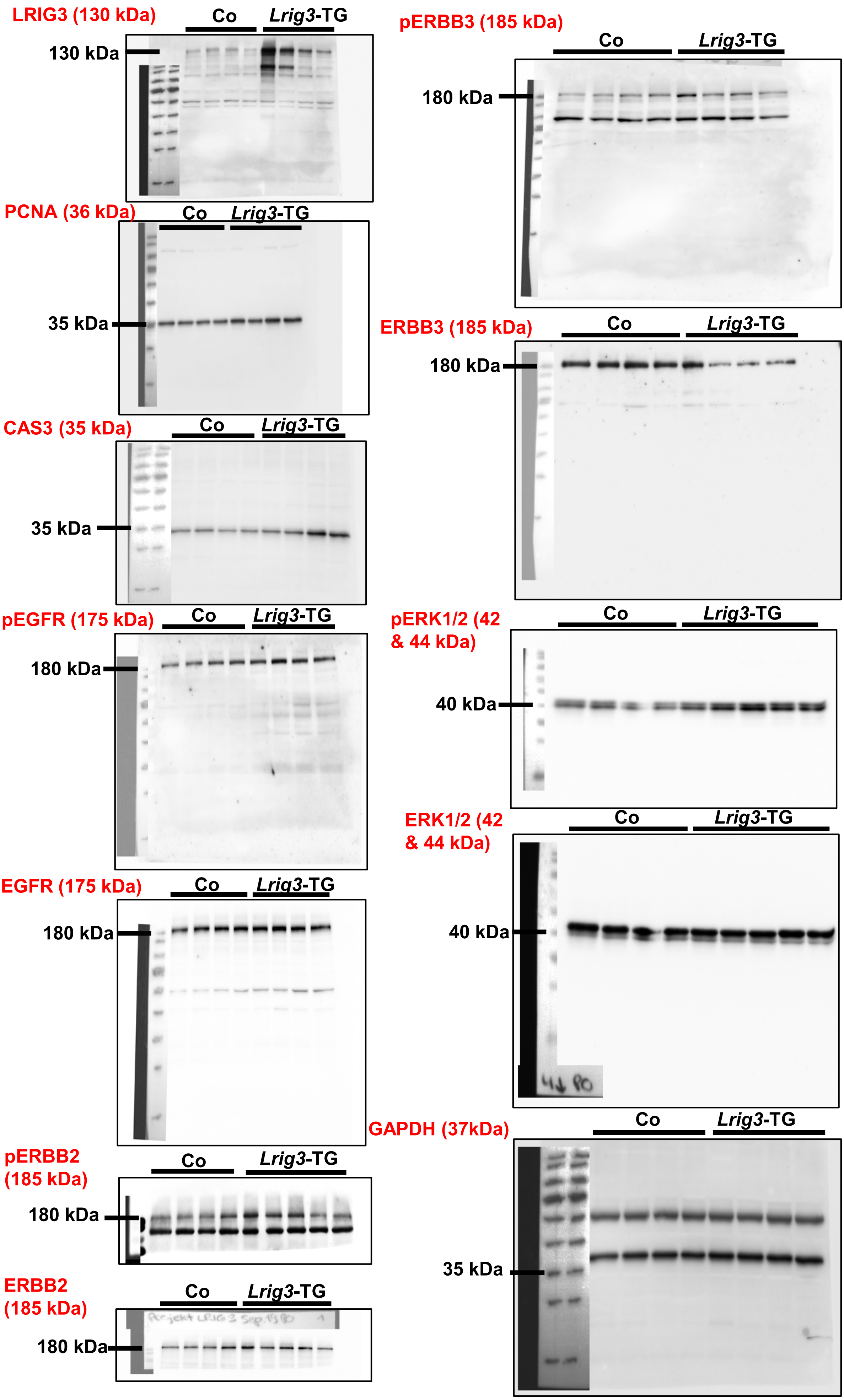

Ghorbanalipoor et al. Fig. S1
