# Supplementary material for: Cutaneous gain-of-function mutation of LRIG3 leads to alopecia by upregulation of ERBB, PI3K/AKT, NOTCH1 signaling pathways
Source: Sci Rep. 2026 May 30;16:27334. doi: 10.1038/s41598-026-54295-5 (PMC13530143; doi:10.1038/s41598-026-54295-5)
Supplement: Supplementary file 7 [file 41598_2026_54295_MOESM7_ESM.docx]

**Supplementary Table S1.** List of antibodies used in this study.

| **Primary antibodies** | **Vendor** | **Host** | **Dilution** | |
| --- | --- | --- | --- | --- |
|  |  |  | **WB** | **IF** |
| LRIG3 | R&D Systems, #AF3495 | Goat | 1:500 | 1:100 |
| LRIG1 | R&D Systems, #AF3688 | Goat | 1:500 | 1:100 |
| LRIG2 | Abcam, #Ab121472 | Rabbit | 1:500 |  |
| PCNA | Cell Signaling, #13110 | Rabbit | 1:1000 |  |
| p-EGFR (Tyr 1068) | Cell Signaling, #2220 | Rabbit | 1:1000 |  |
| EGFR | Cell Signaling, #2232 | Rabbit | 1:1000 |  |
| p-ERBB2 (Tyr 877) | Cell Signaling, #2241 | Rabbit | 1:1000 |  |
| ERBB2 | Cell Signaling, #4290 | Rabbit | 1:1000 |  |
| p-ERBB3 (Tyr 1289) | Cell Signaling, #4791 | Rabbit | 1:1000 |  |
| ERBB3 | Cell Signaling, #12708 | Rabbit | 1:1000 |  |
| p-VEGFR2 (Tyr 1175) | Cell Signaling, #2478 | Rabbit | 1:500 |  |
| VEGFR2 | Cell Signaling, #2479 | Rabbit | 1:1000 |  |
| PDGFRA | Cell Signaling, #3174 | Rabbit | 1:1000 |  |
| PDGFRB | Cell Signaling, #4564 | Rabbit | 1:1000 |  |
| FGFR1 | Cell Signaling, #9740 | Rabbit | 1:1000 |  |
| pERK1/2 (Thr202/Tyr204) | Cell Signaling, #4370 | Rabbit | 1:1000 |  |
| ERK1/2 | Cell Signaling, #9102 | Rabbit | 1:1000 |  |
| pAKT (Ser473) | Cell Signaling, #4060 | Rabbit | 1:1000 |  |
| AKT | Cell Signaling, #4691 | Rabbit | 1:1000 |  |
| p-p38 MAPK (Thr180/Tyr182) | Cell Signaling, #9211 | Rabbit | 1:1000 |  |
| p38 MAPK | Cell Signaling, #9212 | Rabbit | 1:1000 |  |
| p-PI3K p85 (Tyr458)/p55 (Tyr199) | Cell Signaling, #4228 | Rabbit | 1:1000 |  |
| p-PI3K p110G | Cell Signaling, #5405 | Rabbit | 1:1000 |  |
| p-PI3K p110A | Cell Signaling, #4249 | Rabbit | 1:1000 |  |
| PI3K-Class III | Cell Signaling, #3358 | Rabbit | 1:1000 |  |
| p-PTEN | Cell Signaling, #9554 | Rabbit | 1:1000 |  |
| PTEN | Cell Signaling, #9552 | Rabbit | 1:1000 |  |
| HES1 | Cell Signaling, #11988 | Rabbit | 1:500 |  |
| CDH1 | Cell Signaling, #14472 | Mouse | 1:1000 |  |
| KRT1 | BioLegend # 905602 | Rabbit | 1:1000 |  |
| KRT5 | BioLegend # 905504 | Rabbit |  | 1:500 |
| KRT6 | BioLegend # 905701 | Rabbit | 1:1000 | 1:500 |
| KRT10 | BioLegend #905401 | Rabbit | 1:1000 | 1:500 |
| KRT14 | BioLegend #905304 | Rabbit | 1:1000 |  |
| LOR | BioLegend #905104 | Rabbit | 1:1000 | 1:500 |
| IVL | BioLegend #924401 | Rabbit | 1:1000 |  |
| FLG | BioLegend #905804 | Rabbit | 1:1000 |  |
| TGM1 | Proteintech, #12912-3-AP | Rabbit | 1:1000 |  |
| BLIMP1 | Cell Signaling, #9115 | Rabbit | 1:1000 |  |
| LGR6 | Epitomics, #5540-1 | Rabbit | 1:1000 |  |
| P63 | Cell Signaling, #39692 | Rabbit | 1:1000 |  |
| NOTCH1/NICD | Cell Signaling, #3608 | Rabbit | 1:1000 |  |
| RBPJ | Cell Signaling, #5313 | Rabbit | 1:1000 | 1:100 |
| CMYC | Cell Signaling, #5605 | Rabbit | 1:1000 |  |
| CYCD1 | Cell Signaling, #2978 | Rabbit | 1:1000 |  |
| TUBB5 | Cell Signaling #2128 | Rabbit | 1:1000 |  |
| TUBA1A | Cell Signaling #2125 | Rabbit | 1:1000 |  |
| ACTB | Cell Signaling #4970 | Rabbit | 1:1000 |  |
| VCL | Santa Cruz sc-25336 | Mouse | 1:500 |  |
| GAPDH | Cell Signaling, #2118 | Rabbit | 1:1000 |  |
| Rabbit IgG isotype control | Cell Signaling, #3900 | Rabbit |  |  |
| Goat IgG isotype control | Invitrogen, #02-6202 | Goat |  |  |

| **Secondary Antibodies** | **Vendor** | **Dilution** | **Application** |
| --- | --- | --- | --- |
| Alexa Fluor 488 AffiniPure Donkey Anti-Rabbit IgG (H+L) | Jackson ImmunoResearch, #711-545-152 | 1:1000 | IF |
| Alexa Fluor 488 AffiniPure Donkey Anti-Goat IgG (H+L) | Jackson ImmunoResearch, #705-545-147 | 1:1000 | IF |
| Alexa Fluor 647 AffiniPure Donkey Anti-Rabbit IgG (H+L) | Jackson ImmunoResearch, #711-606-152 | 1:1000 | IF |
| Goat-Anti-Rabbit Immunoglobulin G/HRP | Cell Signaling, # 7074 | 1:5000 | WB |
| Horse-Anti-Mouse Immunoglobulin G/HRP | Cell Signaling, # 7076 | 1:5000 | WB |
| Rabbit anti-Goat IgG, HRP | Invitrogen, # 81-1620 | 1:5000 | WB |
